# A question of dissemination: Assessing the practices and implications of research in tropical landscapes

**DOI:** 10.1007/s13280-018-1056-5

**Published:** 2018-04-24

**Authors:** Anne H. Toomey, María Eugenia Copa Alvaro, Matthew Aiello-Lammens, Oscar Loayza Cossio, Jos Barlow

**Affiliations:** 1 Department of Environmental Studies and Science, Pace University, 41 Park Row, #721B, New York, NY 10038 USA; 2 Colección Boliviana de Fauna, Calle 26 de Cota Cota (Ovidio Suárez) Casilla Nº 8706, La Paz, Bolivia; 3 Madidi-Tambopata Landscape Conservation Program, Wildlife Conservation Society, Casilla 3-35 181 SM, La Paz, Bolivia; 4 Lancaster Environment Centre, Lancaster University, Bailrigg, Lancashire LA1 4YQ UK

**Keywords:** Conservation management, Knowledge exchange, Local perceptions, Research-action, Research ethics, Research-implementation

## Abstract

Current debates in the conservation sciences argue for better integration between research and practice, often citing the importance of the diffusion, dissemination and implementation of scientific knowledge for environmental management and policy. This paper focuses on a relatively well-researched protected area (Madidi National Park) in Bolivia in order to present different interpretations and understandings of the implications and availability of research findings. We draw on findings from quantitative and qualitative methods to determine the extent to which research carried out in the region was disseminated and/or implemented for management actions, and to understand subsequent implications for how local actors perceive the value of research and its role in management and conservation. We discuss the critical consequences of these findings for the future of conservation science and practice in biologically and culturally diverse landscapes, with an explicit call to action for academic institutions to support researchers in developing appropriate dissemination strategies.

**Electronic supplementary material:**

The online version of this article (10.1007/s13280-018-1056-5) contains supplementary material, which is available to authorized users.

## Introduction

The maintenance of biodiversity and ecosystem functions in tropical regions is a key conservation challenge that depends in large part on the degree to which human-modified landscapes can be sustainably managed (du Toit et al. 2004; Boreux and Born 2009; Gardner et al. 2009). However, the role of scientists and researchers in contributing to improved management practices has increasingly come under question (Balmford and Cowling 2006; Knight et al. 2008; Arlettaz et al. 2010). It is often argued that although much research has implications for management, there is a “mismatch… between the ecological knowledge generated by researchers and that applied by practitioners” (Hulme 2014, p. 1131). This may be compounded by the frequent failure of researchers to communicate the findings of their work with local stakeholders—particularly with people who live in the regions under study (Knight et al. 2008; Sunderland et al. 2009; Gossa et al. 2014).

Previous literature highlights the potential for conservation gains created as a result of improving integration of research and management, addressing the difficulty of non-academics to access and understand relevant scientific information, and the tendency for conservation scientists to prioritize publishing in top tier journals over engaging more directly in conservation interventions (Meijaard and Sheil 2007; Pullin and Knight 2009; Shackleton et al. 2009; Arlettaz et al. 2010; Gossa et al. 2014). However, research on this topic has generally focused on the extent to which published research in conservation journals has led to (or has the potential to lead to) uptake of management recommendations among practitioners (e.g. Flaspohler et al. 2000; Matzek et al. 2013; Gossa et al. 2014; Walsh et al. 2014). To our knowledge, there has been no look at actual rates of diffusion, dissemination and implementation as reported by researchers themselves. In particular, there is a lack of research on the relationship between the scale(s) at which researchers perceive their research has implications for management, and subsequent knowledge-exchange strategies as engaged in by researchers. In addition, there is a lack of understanding with regard to how people living and working in the regions where research is conducted, such as indigenous communities, protected area staff, and/or extension agents, perceive the extent to which such research is disseminated and/or implemented (Toomey 2016). This is an important omission, as it is widely acknowledged that natural resources governance happens at multiple levels of scale (local, regional, national and international), requiring knowledge to be made accessible to decision-makers operating at different levels (Berkes 2007; Ostrom 2010). In cash-strapped parts of the world, where there are limited resources for federal enforcement of national environmental policies, local and/or regional governance structures can be extremely important for natural resources management (Shanley and Laird 2002; Kainer et al. 2009). Thus, it is necessary for conservation researchers working in these contexts to take a polycentric view of governance, in which management occurs at multiple levels, and to develop dissemination strategies accordingly (Duchelle et al. 2009).

This paper advances the debate by conducting quantitative and qualitative research on how different forms of research diffusion, dissemination and implementation are reported and perceived by different stakeholder groups in a relatively well-studied protected area in Bolivian Amazonia. We ask the following questions: (1) In what ways and at what levels was the research conducted in the region relevant for management? (2) To what extent were research results made accessible to those who could use the information? (3) Does this differ depending on whether the PIs were based at Bolivian or foreign institutions? We then discuss our findings within the wider context of the international literature on science communication and knowledge co-production. Finally, we discuss assumptions mentioned in the literature on research dissemination and implementation, and provide recommendations for ways in which academic institutions can support researchers in this regard.

## Materials and methods

### Study site

**Fig. 1:**
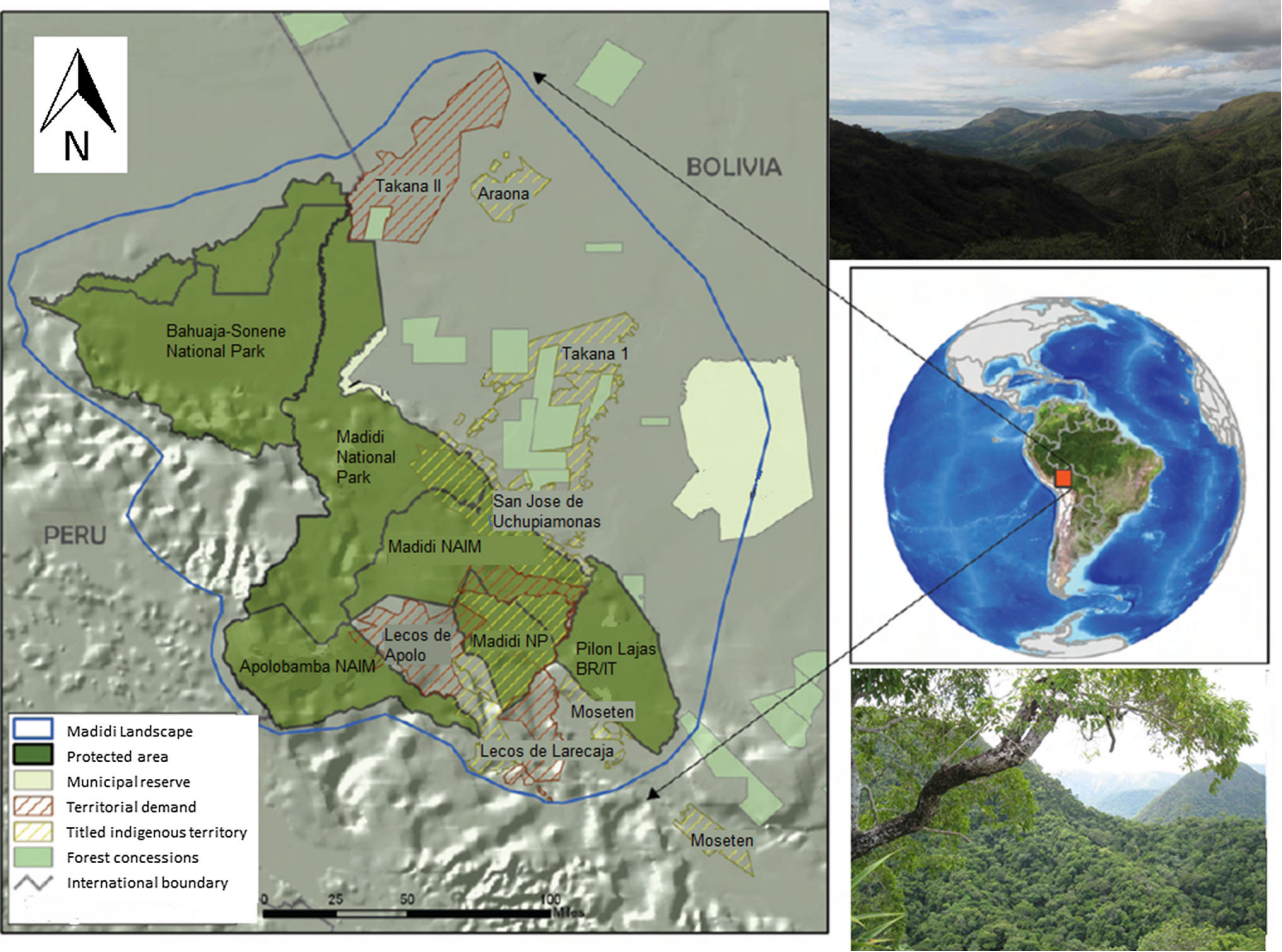
Map and images of Madidi landscape. Map copyright: Wildlife Conservation Society Bolivia, photos by Anne H. Toomey

Madidi National Park and Natural Area of Integrated Management (Madidi NP/NAIM) incorporates approximately 19 000 km^2^ across the tropical Andes in the northwest of the La Paz department in Bolivia. It is one of the most biologically diverse protected areas on the planet, where 8244 species of vascular plants, 182 mammal species and 917 species of birds have been formally registered (SERNAP 2012; Hawes et al. 2015). The park is also notable for its cultural diversity, and the communities located within and adjacent to Madidi NP/NAIM consist of multi-ethnic groups, including lowland indigenous peoples native to the region (Takana, Tsimane’-Mosetén, Leco) and highland indigenous populations (Aymara and Quechua) that have arrived in recent decades (Bottazzi 2008). Four indigenous territories (TCOs) overlap the protected area (San José de Uchupiamonas, Takana 1, Lecos de Apolo and Lecos de Larecaja), and two others border Madidi (Takana II and the Tsimane’-Mosetén, located in the neighbouring Pilón Lajas Biosphere Reserve) (Fig. 1).

### Systematic analysis of past research

We carried out a systematic analysis of all scientific research conducted between 2004 and 2013 in Madidi NP/NAIM. This was done by conducting an exhaustive review of documentation physically located in the Madidi park offices and recording all research permit applications and other references related to scientific research (e.g. technical reports, publications) in a database in November of 2013. We identified a total of 88 research projects (excluding the research described in this paper). For all projects, we recorded the following information (to the extent that it was available): principal investigator(s) (PIs), institution(s), type of study, length of data collection, title of project, subject, geographical location, research objectives and contact information of the PIs. Over a 1-year period (between December 2013 and November 2014) the primary author then attempted to follow up with the PIs listed on each project to verify the information obtained and ask additional questions (in English and Spanish) about the research, such as: Did the research have any implications for management and/or policy? (What were they?) Were the research results disseminated? (How?) Were the research results published? (Where?) Did the research lead to any management decision or action? We omitted details that could potentially identify specific research projects in order to protect the anonymity of the PIs queried.

### Interviews and workshops

In addition to the systematic analysis of previous research, between 2012 and 2014, we carried out additional qualitative data collection with actors living and working in the region to discuss perceptions regarding the importance of scientific research, the extent to which it is locally disseminated, and its relevance for the management of natural resources in the region. These methods included 91 unstructured and semi-structured interviews, 38 with indigenous people (leaders and community members residing in nine communities in the Madidi region), 25 with park staff (park guards, directors and project leaders), 21 with scientists working in the region and 7 with government officials or staff from other NGOs. Questions and the discussions (and duration) varied according to the situation and interviewee, but a sample interview schedule is provided in Appendix S1.

We also held workshops with all of the above stakeholders to discuss perceptions regarding the past, present and future of scientific research in the region. These were designed to discuss to what extent previous research had been disseminated/implemented for management, personal experiences with research, and about the relevance of research in general. A total of 30 park guards from Madidi and Pilón Lajas participated in 3 workshops at protected area offices in 2013. Workshops were also held in two indigenous communities (San José de Uchupiamonas and San Miguel) that had hosted many researchers in the past, and were organized around a proposal to create community norms to negotiate their relations with researchers in the future. Two ‘communication and dissemination training’ workshops were also held with 40 + students and staff of the National Herbarium (a botanical institute) in La Paz. Workshops were audio recorded and photographed with the informed consent of participants (See Appendix S2 for more details on methodology). Official ethical approval for the research described above was obtained in June 2012 from the Lancaster University Research Ethics Committee, and names and identifying details were removed to ensure anonymity for all respondents.

### Quantitative analysis

We identified contact details of PIs for 75 of the 88 research projects identified in the database. Three projects were excluded from analysis because they were determined to be government-led evaluations that did not have research as the primary aim (e.g. hydrological measurement, environmental impact assessment). We then contacted all PIs either in person, by telephone or via email. Of these contact attempts, 15 went unanswered, one person explicitly refused to provide requested information, 11 responded that the study in question was not carried out in Madidi due to permitting problems or other issues, and nine responded by email but did not complete the questions. For the remaining 40 studies, complete information was obtained directly from the PIs. The results presented below are based on this subsample of projects.

For our statistical analysis, we applied Fisher’s exact test to examine whether there were significant associations between research project characteristics (i.e. investigator nationality, scope of research implications, and scope of dissemination). We assumed a two-sided alternative hypothesis, as we had no a priori assumptions regarding the direction of potential associations. We chose this over a *χ*^2^ test because the latter performs poorly when some cells of the contingency table have values of five or less, a condition that frequently applied to our data. Additionally, we applied a generalized linear model (GLM) with a log link function, assuming a Poisson distribution for the residuals, and considering the number of research projects as a response variable. This model form is an alternative to the application of contingency tables, with the goal of examining associations between categorical variables (Quinn and Keough 2002; Logan 2010). For predictor variables, we considered nationality (national versus international), whether the research had local/regional implications, and whether the research was locally disseminated. The goal with the statistical analysis was to examine whether there were associations between these three research project characteristics. These analyses were performed using the R statistical environment (R Core Development Team 2013).

### Qualitative analysis

Conservation scientists are increasingly interested in social science research, but they often have difficulty interpreting this research because of the different types of principles and assumptions underlying quantitative and qualitative data collection and analysis (Drury et al. 2011; Moon and Blackman 2014). While in quantitative data, especially in the natural sciences, there is an established norm towards objectivity, the ‘subjective’ plays an important role in qualitative social science research, which seeks to uncover the complex ways that individuals and social groups experience and understand the world. We conducted our qualitative analysis with the use of the coding software Atlas.ti. version 7.1.8 (2014), a computer-assisted qualitative data analysis software program. We used both a priori and open coding in order to connect themes sought out through interview and workshop questions, as well as to allow additional unanticipated patterns emerge from the data (Kelle 2007; Saldaña 2015).

### Defining dissemination

In the conservation science literature on the space between research and implementation, the terms ‘diffusion’, ‘dissemination’ and ‘implementation’ are frequently used, but rarely defined (Flaspohler et al. 2000; Sunderland et al. 2009). However, within the evidence-based medical literatures attempts have been made to clarify meanings of these terms, which are often used interchangeably when comparing passive and active forms of scientific communication. In our analysis, we group diffusion, dissemination and implementation activities according to the definitions in Table 1, and provide corresponding examples. In the questions sent to PIs, ‘dissemination’ was not explicitly defined, but rather was left open to interpretation in order to better understand the spectrum of activities that researchers listed under this term. Subsequently, in our analysis, we grouped diffusion, dissemination and implementation activities according to the definitions in Table 1, and provide corresponding examples from the conservation sciences.

**Table 1:** Differences between terms ‘diffusion’, ‘dissemination’, and ‘implementation’

Term	Definition
Diffusion	Diffusion is a passive process, whose “slow and deliberate pace” is valued in science because of assumptions about the importance of validating and replicating data before advocating for knowledge application (Kerner and Hall 2009, p. 520). “Light diffuses from a source; it is not targeted; it is haphazard; it is largely unplanned and uncontrolled. Those who receive diffused messages were likely already open to and seeking out the message” (Lomas 1993, p. 226). Examples of this in the conservation sciences include publishing in academic journals and presenting scientific results at international conferences
Dissemination	Dissemination is considered to be a more proactive process, in which the information is directly targeted and tailored to those who might use the information. The general goal of dissemination is increased awareness, especially among those who would not normally actively seek out the information otherwise. Examples of this in the conservation sciences include providing reports to policymakers and giving presentations on research to local actors
Implementation	Implementation goes beyond the informational goals of both diffusion and dissemination to overcome barriers to putting given knowledge into practice. This involves both the explicit stating of the implications of the information as well as the acknowledgement of additional conditions (social, organizational, behavioural) that could put constraints on the application of generated information. It is considered to be a local process of communication in which the research findings are only part of the decision-making process (Lomas 1993). Examples of this in the conservation sciences include the incorporation of research findings into protected area management plans, and actions taken based on the research

## Results

In this section, we discuss the quantitative results of the systematic analysis separately from the qualitative results gathered through interviews and workshops, due to differences in data collection and analyses. We further integrate these results in our “Discussion” section.

### Quantitative results

Of the total number of projects in the database (*N* = 88), 46% were led by PIs based at foreign institutions and 54% were Bolivian based, but due to lower rates of response from foreign-based researchers to our query, below we report on a subsample that includes 35% foreign-based (*n *= 14) and 65% Bolivian-based (*n *= 26) projects. Thirty-eight of the 40 studies (95%) were related to conservation, ecology and/or natural resources management. These included four projects that looked into the social perceptions of conservation strategies and protected area management held by communities and local actors in the region. Twenty-five percent were studies done as part of academic degree program (undergraduate, Masters or doctoral level), with the remaining 75% consisting of academic, institutional and collaborative research. We were not able to further differentiate between research projects as being pure or applied, or NGO-run or academic, as more than half of the research projects were run as collaborations between multiple institutions (frequently NGO–university partnerships) and often had multiple aims, making it problematic to distinguish between research projects focused primarily on publications versus projects with actionable goals in mind.

#### In what ways and at what levels was the research conducted in the region relevant for management?

We asked PIs to indicate whether they perceived that their research had implications for management, and if so, to describe how. Thirty-three of 40 projects (83%) in the systematic analysis stated in the affirmative that their project had definite or potential implications for management. Respondents described multiple ways in which findings from their respective studies could be utilized in management decisions, and a greater number of the open-ended responses specified locally focused (community-level or park-level, 35 and 70%, respectively) over broader implications (national or global, 45 and 10%, respectively) (see Table 2).

**Table 2:** Local, regional, national and international management implications of research. Responses to the question, “Does your research project have implicit or explicit implications for management,” and to a follow-up question, “What are they?” Responses in the affirmative were analysed for specific reference to the geographical level to which the management implications were directed (as often multiple levels were identified total percentages added up exceed 100%)

Scale of management implication (local to global)	Number of projects that mentioned	% (*n *= 40)	Examples from dataset
Community-level	14	35	Communicating the importance of interspecies dynamics so local communities can better understand impacts of hunting (and develop adaptive strategies such as hunting zones) Informing people about pathogenic relationships with regard to disease transmission between domestic and wild animals Providing new economic opportunities and improving existing strategies for people living within and adjacent to protected areas
Specific to local region (Madidi NPNAIM and surrounding municipalities)	28	70	Contributing to park management plans by providing information of species inventories and other biophysical information Understanding local effects of climate change in order to develop adaptation strategies Giving recommendations to park staff for how to improve local peoples’ perceptions of the protected area management
National/Regional	18	45	Maintaining connectivity with other protected areas in the region (conservation corridors) for large mammal protection Upholding constitutional commitments to integrated management of forest resources in the context of reciprocity and equality Identifying conservation gaps and proposing changes to the national protected area system to improve conservation of certain threatened taxa
International	4	10	Knowing about the spatial requirements of certain species Demonstrating the impacts of fire and deforestation on certain ecosystems and taxa Providing information for the IUCN global assessments
No implications	7	18	

#### To what extent were research results made accessible to those who could use the information?

We asked researchers to report on whether they diffused or disseminated research results, and to specify the method of knowledge exchange. We found that diffusion strategies were the most common type of knowledge exchange reported by researchers, with 45% of PIs having published research results in international academic literature, 30% in Bolivian or regional (specific to South America) journals, and 13% at international or national conferences. Dissemination practices related to popular press and grey literature, such as blog posts, government reports and/or press releases, were somewhat less prevalent, with 10% of researchers reporting having engaged in these strategies at the international level, and 20% at the national level. With regard to more locally focused knowledge-exchange strategies, a total of 12 projects (30%) involved dissemination of written materials to local actors, such as community leaders and/or protected area administration. Nine projects (23%) additionally engaged in oral dissemination practices, such as workshops, meetings and/or presentations of research with local actors.

#### Does the extent to which the research results were made accessible to those who could use the information differ depending on whether the PIs were based at Bolivian or foreign institutions?

Considering whether the PI was based at a Bolivian (65%, *n *= 14) or foreign (35%, *n *= 26) institution, we found a highly significant association as to whether the research was diffused and/or disseminated at the local, regional, or national level versus the international level (Fisher’s exact test, *p* = 0.0002). With regards to diffusion, more foreign-based researchers published in international journals or presented at international conferences compared to those that were Bolivian based (86 and 35%, respectively); while the inverse was true for national or regional publications and/or conferences, where 42% of Bolivian-based researchers but only 14% of internationally based researchers contributed. Regarding dissemination, only 1 foreign-based research project engaged in any form of local, regional or even national knowledge exchange. Whereas among Bolivian researchers, 31% published in the national grey literature, 42% handed written materials over to local actors, and 31% presented results orally through workshops or presentations. With regards to implementing research findings, only 14% of projects in total fell into the two categories described here, and all were carried out by researchers based in Bolivian institutions (see Table 3).

**Table 3:** Responses regarding research diffusion, dissemination and implementation. Combined responses to questions: Were the research results disseminated? (How?) Where the research results published? (Where?) Did the research lead to any management decision or action? The percentages do not add up to 100% as several projects engaged with multiple strategies

Type of strategy	Specific strategy	Foreign *n *= 14		Bolivian *n *= 26		Total *n *= 40	
		*n*	%	*n*	%	*n*	%
None	No dissemination/unsure/left blank	2	14	0	0	2	5
None yet	Dissemination phase planned but not yet begun	2	14	4	15	6	15
Diffusion	Publication in international journal/thesis in international	10	71	8	31	18	45
Diffusion	Publication in national/regional journal/thesis in national university	2	14	10	38	12	30
Diffusion	Presentation in academic conference or seminar—international	2	14	3	12	5	13
Diffusion	Presentation in academic conference or seminar—national/regional	0	0	5	19	5	13
Dissemination	Grey literature publication (blog, report, press release, etc.)—international	2	14	2	9	4	10
Dissemination	Grey literature publication (blog, report, press release, etc.)—national	0	0	8	31	8	20
Dissemination	Handed written material over to local actors (park staff, community leaders)	1	7	11	42	12	30
Dissemination	Workshops/presentations/meetings in Madidi region with local actors	0	0	9	35	9	23
Implementation	Was included in national, regional or community management plan	0	0	4	15	4	10
Implementation	Directly led to local conservation/mgmt. actions	0	0	2	8	2	5

#### Cross-comparison of factors

**Fig. 2:**
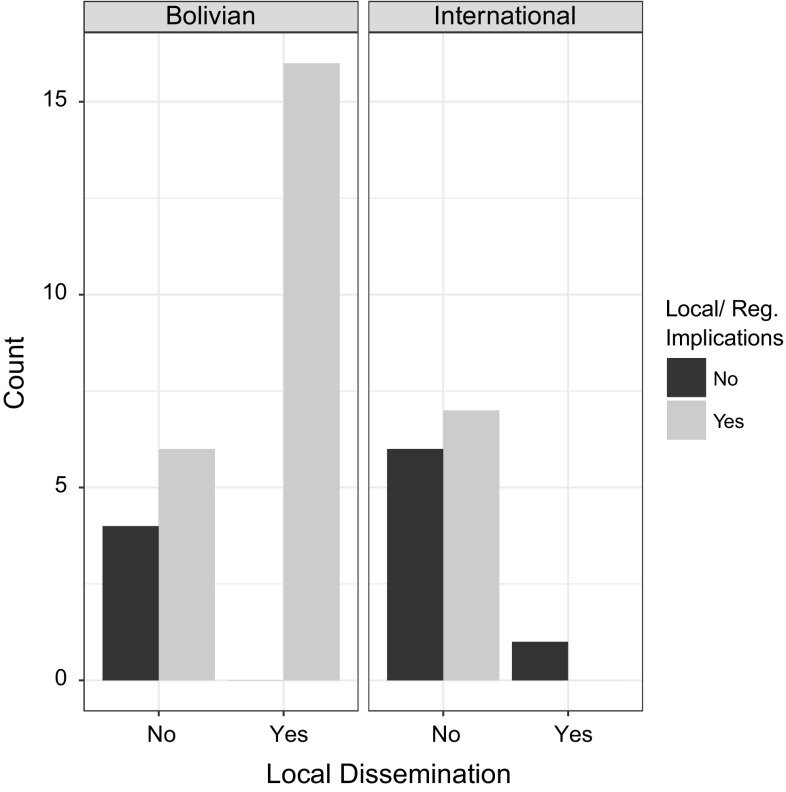
Relationship between local dissemination rates and whether PIs reported that their research had local or regional implications for management. The graph to the left represents the implications–dissemination interaction among Bolivian researchers and the graph to the right shows the interaction among foreign researchers

We also sought to determine whether there was a statistically significant relationship between the scales at which researchers indicated that their research had implications for management and the knowledge-exchange types in which they engaged. While we did identify a significant association between whether researchers claimed that their work had implications for local or regional resource management and whether they disseminated their work locally (Fisher’s exact test, *p* = 0.012), this association appears to be mostly driven by the fact that those researchers who stated that their research did not have local or regional implications also did not locally disseminate their research at those same levels of scale (91%, *n *= 11). By comparison, those who did assert local or regional implications were more evenly split on whether they shared information at a local or regional level, 55% (*n *= 29) having done so (Fig. 2).

**Fig. 3:**
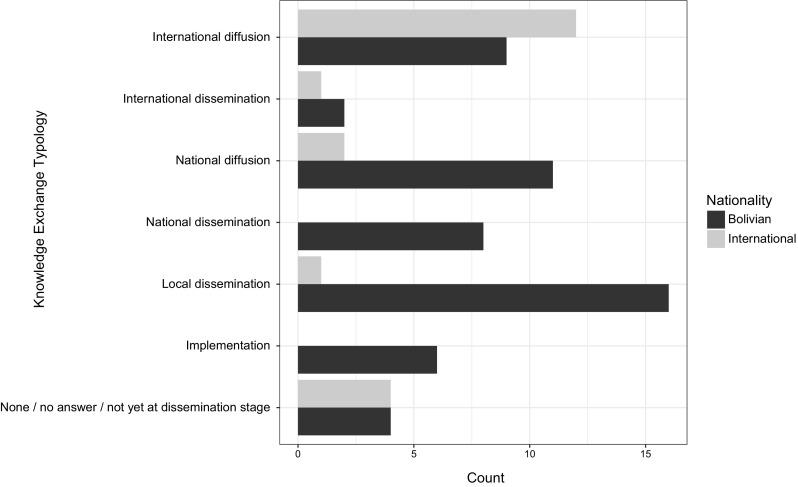
Diffusion and dissemination of research by Bolivian and foreign-based researchers. *n *= 40

In examining the three-way relationship between nationality, local or regional implications, and local dissemination via the GLM analysis, we found additional evidence for interaction effects (Fig. 3). Based on model selection via analysis of deviance, the GLM that included a three-way interaction was a better fit than either models with no interactions among the predictor variables or those that consider joint independence of nationality (i.e. dependence among implications and dissemination, but independent of nationality). This implies that there is a relationship between whether the researcher was Bolivian or foreign, whether their research had implications at the local/regional level, and whether they were inclined to disseminate locally. This finding was further supported by the results of Fisher’s exact tests of the association between local or regional implications and local dissemination, done separately for national versus international scientists (*p* = 0.0145 and *p* = 1, respectively). The large differences in *p* for these two tests suggests an interaction effect of nationality, though sub-setting our data set to perform these two tests results in relatively low sample sizes, and increasing this sample size would further resolve these interaction effects.

### Qualitative results

Our qualitative analysis resulted in 21 different codes representing 433 quotations (see Appendix S3 for code list and frequency counts). Based on the frequency and perceived importance of quotations categorized under each code, three of these codes were selected for closer analysis: Local perceptions of research (*n *= 64), Diffusion and Dissemination (*n *= 56), and Results/Benefits (*n *= 34). Quotation outputs were generated for these codes and further analysed for patterns to develop the qualitative results section below (also see Appendix S3 for code frequency counts).

#### In what ways and at what levels was the research conducted in the region relevant for management?

Interviews and workshops revealed mixed perceptions among participants with regard to the relevance of research conducted in the region for management. On one hand, the potential value of scientific research in providing knowledge and recommendations for management at local, regional and national scales of governance was discussed by all actors. Some interviewees stressed that technical information provided through applied research and monitoring could be used to gauge the effectiveness of management interventions and to track environmental change over temporal and spatial scales. Actors who lived in the region, such as indigenous community members, expressed strong interest in learning about the results of previous and existing scientific research on their lands. Biological information was of particular interest to many, as people in the region were concerned with the health of the animals and resources they depend upon for their livelihoods. Concerns over water quality and availability, the health of populations of endangered fauna, and pollution from economic activities were commonly mentioned as important research topics by community members and protected area staff (see Table 4).

**Table 4:** Research topics of interest and relevance to local actors. This list is a compilation of the top priority research topics and was generated through workshops with local actors (park rangers and communities) in PNANMI Madidi and surrounding region

Research topic	Priority for administration of protected areas	Priority for other social actors (indigenous communities)
Studies of water quality (levels of mercury and other heavy metals, the level of water pollution, maintenance of slopes)	√	√
Environmental and social impacts of mining	√	
Potential impacts of proposed megaprojects (oil exploration, hydroelectric dams)	√	√
Ecosystem services provided by the protected area	√	
Impacts of climate change on ecosystems and melting of glaciers in high altitude zones of park	√	√
Population density and productive capacity studies for animals typically consumed in overlapping indigenous territories (and in buffer zone)	√	√
Local awareness regarding the laws and management of the protected area	√	
Studies of flora and fauna, involving local guides (for tourism purposes)	√	√
Traditional medicine and ancestral uses of flora and fauna, including spiritual healing practised in communities	√	√
Cultural history of communities and the continuity of indigenous cultures	√	√
Social and environmental impacts of tourism	√	
Census of timber species, distribution and abundance	√	√
Study of importance of the social participation of the management committee (to what extent they support the management of the protected area)	√	

However, many researchers who were interviewed argued that in the current political climate in Bolivia, technical information is not highly valued by decision-makers. Rather, many respondents perceived that decisions are more typically based on politics and local knowledge and that research needed to be more “practical” than “technical” if it were to be able to be implemented. In this context, it was suggested that the role of national universities has traditionally been less to provide new knowledge than to train and educate young researchers.

Some respondents additionally argued that it is not always possible to know what the impact of research will be beforehand, and thus that even “pure” research with no applied aims could potentially have important implications for land management and natural resources. However, it was also pointed out that this lack of clear differentiation between what is ‘pure’ research and what is applied can lead to confusions and frustrations in communities in which research is conducted, but does not have immediate local value.

#### To what extent were research results made accessible to those who could use the information?

There was general agreement among in all workshops and interviews conducted that there was a gap between the amount of information conducted and the extent to which it was made available to decision-makers. However, different explanations for this issue were put forward. Some researchers emphasized that the problem was primarily due to lack of capacity among decision-makers in Bolivian society to manage and make use of such information. They emphasized that while there is a great deal of knowledge available on the internet and in books, such information is not typically sought out—some referenced a lack of reading in the culture. A few researchers emphasized that they had handed over reports to local stakeholders multiple times, but there was a need to systematize the information that had already been gathered, in order to avoid the common problem of duplicating studies.

Many respondents indicated that the lack of dissemination was a central failing of the research community. Some interviewees pointed to research carried out for institutional purposes, which once completed, was filed away into the bookshelves of that particular institution and not shared more broadly. Professional and institutional “envy” or “guardedness” was also cited as a central reason for which research was not more adequately disseminated, in part due to limited financial resources available to support research.

People living within the Madidi region frequently mentioned the missed opportunities in not knowing about the results of research conducted on their lands. Park guards lamented the lack of such information to be able to inform local communities about the status of their natural resources. They considered this a missed opportunity due to the potential synergy of impact when existing local observations are corroborated with technical information. Some indigenous leaders expressed frustration that research results are published in languages and in literature inaccessible to them. Brainstorming during the various workshops offered many creative suggestions for how scientists could better communicate the results of their work such as collaboration with local communities and organizations when choosing a research topic to ensure it is of mutual interest (i.e. co-created research agendas), providing opportunities for local participation at different stages of the research, and finding ways to share results that can reach larger segments of the population (i.e. educational videos or presentations at community meetings). Interviews with researchers pointed more specifically to lack of training in the importance of knowledge exchange and the difficulty in knowing what to share that would be of use beyond academic circles.

#### Does the extent to which the research results were made accessible to those who could use the information differ depending on whether the PIs were based at Bolivian or foreign institutions?

Differences in dissemination rates among foreign versus Bolivian-based researchers was not something that was commonly discussed in interviews or workshops, and there was little qualitative evidence that people living in the region differentiated between types of researchers based on nationality or discipline. Rather, our qualitative analysis found that issues of dissemination and implementation were experienced with and by researchers of all backgrounds. In some cases misinterpretation of the objectives and outputs of scientific research led to misunderstandings and unmet expectations on the part of host communities, and both national and foreign researchers said that they were increasingly subjected to requests and demands that were seemingly unrelated to their proposed research.

However, one notable difference between foreign and Bolivian-based researchers that emerged from our qualitative data was a greater lack of awareness of and preparedness among non-nationals to deal with the social and political components of the research context in Bolivia. In particular, foreign researchers who were conducting research for graduate studies mentioned lack of understanding and support from supervisors in their home institutions with regard to questions about research dissemination and relevance posed to them by local stakeholders.

## Discussion

Comparing and contrasting the different findings based on our quantitative and qualitative data reveal both areas of overlap as well as discrepancies. On the one hand, our systematic analysis of 10 years of research carried out in Madidi found both significant implications of the research for decision-making, in particular at local, regional and national levels of scale. On the other hand, we found that local and regional dissemination practices were only practised by a minority of researchers (30%), and this was especially the case for foreign-based researchers. Our qualitative data additionally presents widely held perceptions among actors living and working in the Madidi region that research was not being used in decision-making because of both limited capacity as well as lack of accessibility to the research results by decision-makers. Our qualitative findings include multiple explanations for this lack of dissemination, which is tied into wider discussions about the role of scientific research in conservation.

The relationship between scientific knowledge and policy is a deeply contested issue in the conservation sciences, and has gained increasing attention in recent years (Milner-Gulland et al. 2012; Adams and Sandbrook 2014; Bertuol-Garcia et al. 2017). While some scholars have advocated for the greater role of scientific evidence in decision-making (Sutherland et al. 2004; Haddaway and Pullin 2013), others have criticized such approaches as being overly linear, and neglecting the importance of two-way modes of knowledge production (Van Kerkhoff and Lebel 2015; Toomey et al. 2017). Our findings demonstrate that these debates are not only taking place in academic circles, but are also of concern and interest to non-scientific actors who live and work in regions where research is carried out.

In the Madidi region during the 1990s and early 2000s, wildlife studies were carried out by biologists in collaboration with indigenous hunters and fishers to gather data on the spatial needs of the local populations, which indigenous leaders then used to support claims for territorial autonomy (see Copa and Townsend 2004). These types of research projects can help to build relationships between different actors with shared interests. This is of current relevance, as the Bolivian government passed a decree in 2015 to allow hydrocarbon exploration within Madidi and other large protected areas in the country, and by doing so, reduce the autonomy previously granted to established indigenous territories. In these contexts, scientific data can help to better understand the environmental and social consequences of such ‘mega projects’, and additionally to strengthen the capacity of conservation organizations and indigenous groups through partnerships (Zimmerer 2006; Painter et al. 2011).

Lack of research dissemination is of practical consequence as well. People living within or adjacent to protected areas are often those who determine who can access their lands and whether research will be permitted or not (Shanley and Laird 2002). This is of particular concern in areas of both high biological and cultural importance, where research longevity is essential to be able to detect important temporal trends in land use, ecosystem functioning, among other changes, and where lack of access to field sites will impede the capacity for such research to be conducted.

## Conclusion and recommendations

Our study explores the implications of diffusion, dissemination and implementation strategies for conservation and future research in the Madidi region and far beyond. The results strengthen arguments put forward in previous literatures about the importance for conservation scientists to actively engage in local dissemination and implementation activities (Ehrlich and Pringle 2008; Arlettaz et al. 2010). What lessons can the conservation science community learn from this? What actions could be taken?

First, we argue that dissemination, not only diffusion, should be seen as an integral part of the research process in the conservation sciences, rather than something to be done after the “real work of academics” is complete (Nadkarni 2004). Empirical research does not support the perceived tradeoff between academic success and engaging with society. Jensen et al. (2008) found that researchers who were the most active in dissemination (such as public outreach through popular media), teaching and extra-academic collaborations also performed better academically than their colleagues who did not engage in such activities. In addition, they identified that the main reason researchers did not participate in public engagement was not primarily due to lack of time or recognition for their careers, as has been often been argued under the mantra of ‘publish or perish’, but rather because they believed that their colleagues did not consider dissemination to be important, and they lacked confidence in their ability to do such work successfully.

These findings point to the need for both formal and informal support for researchers to engage in dissemination and implementation. Dissemination can be very challenging to the inexperienced, is as much of a skill as academic writing, and researchers at all levels need inspiration, training and advice in this regard. Students entering “the field” in the present day face a vastly different terrain than that experienced by researchers in the 1990s or even the early 2000s. The number of degree-granting programs for PhD and master students in environmental studies and science has grown exponentially in the last 20 years, and many of these programs require students to do independent research for the fulfilment of their degree (Clark et al. 2011). Foreign-based research institutions have traditionally viewed tropical regions such as Amazon (and the protected areas within them) as potential laboratories in the field where students can learn how to do research, not always taking into account the complex social, political and cultural dynamics happening within them (Smith 1999; Chilisa 2012). The significance of this is that there are more inexperienced researchers in the field now than ever before, and the history of research in certain regions is longer and more complex, particularly in biologically and culturally diverse regions attractive to researchers and funders. Because of this, senior researchers should prepare students by discussing ideas for how to address questions about the potential implications of their work, and how resulting knowledge will be disseminated, with people living on or adjacent to the land where the fieldwork is being conducted.

More formally, conservation departments and graduate degree programs could take deliberate steps to ensure that dissemination is an integral part of the research process by recognizing the importance of allocating funds and a specific period of time in the research project for carrying it out and communicating that need to funders (Kainer et al. 2006; Duchelle et al. 2009). For example, a ‘dissemination’ phase could be inserted into doctoral research projects, which would additionally provide new researchers with valuable social skills, such as community outreach and policy work, for future research projects in conservation (as an example, see the University of Florida’s Tropical Conservation and Development Program). This would help to ensure that the next generation of conservation leaders is equipped with more than technical expertise to meet the interdisciplinary challenges that current and future global environmental change will require (Cook et al. 2013; Pietri et al. 2013).

However, researchers first need to be clear about the differences between the terms diffusion and dissemination, and clarify which strategies are most relevant for the kind of research they are doing, especially if trying to reach those who might not otherwise seek out the information. Even the “purest” research can have very relevant, direct and local consequences for the management of a given landscape, and applied research may reveal theoretical insights that are not so directly put into action, so different knowledge-exchange strategies will be appropriate for different situations. It may be helpful for those doing research to imagine the legacy of their work on the ground, and to seek to leave the places where they gather their data accessible for future generations of researchers and practitioners, to keep the doors open to collaboration and exchange.

## Electronic supplementary material

Below is the link to the electronic supplementary material.

Supplementary material 1 (PDF 358 kb)

## References

[CR1] Adams WM, Sandbrook C (2014). Conservation, evidence and policy. Oryx.

[CR2] Arlettaz R, Schaub M, Fournier J, Reichlin TS, Sierro A, Watson JEM, Braunisch V (2010). From publications to public actions: When conservation biologists bridge the gap between research and implementation. BioScience.

[CR3] Balmford A, Cowling RM (2006). Fusion or failure? The future of conservation biology. Conservation Biology.

[CR4] Berkes F (2007). Community-based conservation in a globalized world. Proceedings of the National Academy of Sciences.

[CR5] Bertuol-Garcia D, Morsello C, El-Hani CN, Pardini R (2017). A conceptual framework for understanding the perspectives on the causes of the science–practice gap in ecology and conservation. Biological Reviews.

[CR6] Boreux V, Born J (2009). Sharing ecological knowledge: Opportunities and barriers to uptake. Biotropica.

[CR7] Bottazzi P (2008). Linking ‘Socio-’ and ‘Bio-’ Diversity: The stakes of indigenous and non-indigenous co-management in the Bolivian lowlands. Geographica Bernensia.

[CR8] Chilisa B (2012). Indigenous research methodologies.

[CR9] Clark SG, Rutherford MB, Auer MR, Cherney DN, Wallace RL, Mattson DJ, Clark DL, Foote L (2011). College and university environmental programs as a policy problem (part 1): Integrating knowledge, education, and action for a better world?. Environmental Management.

[CR10] Cook CN, Mascia MB, Schwartz MW, Possingham HP, Fuller RA (2013). Achieving conservation science that bridges the knowledge–action boundary. Conservation Biology.

[CR11] Copa ME, Townsend WR (2004). Aprovechamiento de la fauna por dos comunidades tsimane: un subsidio del bosque a la economia familiar. Revista Boliviana de Ecologia y Conservacion Ambiental.

[CR700] Drury, R., K. Homewood, and S. Randall. 2011. Less is more: The potential of qualitative approaches in conservation research. *Animal Conservation* 14: 18–24.

[CR12] du Toit JT, Walker BH, Campbell BM (2004). Conserving tropical nature: Current challenges for ecologists. Trends in Ecology & Evolution.

[CR13] Duchelle AE, Biedenweg K, Lucas C, Virapongse A, Radachowsky J, Wojcik DJ, Londres M, Bartels W-L (2009). Graduate students and knowledge exchange with local stakeholders: Possibilities and preparation. Biotropica.

[CR14] Ehrlich PR, Pringle RM (2008). Where does biodiversity go from here? A grim business-as-usual forecast and a hopeful portfolio of partial solutions. Proceedings of the National Academy of Sciences.

[CR15] Flaspohler DJ, Bub BR, Kaplin BA (2000). Application of conservation biology research to management. Conservation Biology.

[CR16] Gardner TA, Barlow J, Chazdon R, Ewers RM, Harvey CA, Peres CA, Sodhi NS (2009). Prospects for tropical forest biodiversity in a human-modified world. Ecology Letters.

[CR17] Gossa C, Fisher M, Milner-Gulland EJ (2014). The research–implementation gap: How practitioners and researchers from developing countries perceive the role of peer-reviewed literature in conservation science. Oryx.

[CR18] Haddaway N, Pullin AS (2013). Evidence-based conservation and evidence-informed policy: A response to Adams & Sandbrook. Oryx.

[CR19] Hawes M, Ling R, Dixon G (2015). Assessing wilderness values. International Journal of Wilderness.

[CR20] Hulme PE (2014). Bridging the knowing–doing gap: Know-who, know-what, know-why, know-how and know-when. Journal of Applied Ecology.

[CR21] Jensen P, Rouquier JB, Kreimer P, Croissant Y (2008). Scientists who engage with society perform better academically. Science and Public Policy.

[CR22] Kainer KA, DiGiano ML, Duchelle AE, Wadt LHO, Bruna E, Dain JL (2009). Partnering for greater success: Local stakeholders and research in tropical biology and conservation. Biotropica.

[CR23] Kainer KA, Schmink M, Covert H, Stepp JR, Bruna EM, Dain JL, Humphries S (2006). A graduate education framework for tropical conservation and development. Conservation Biology.

[CR24] Kelle U (2007). “Emergence” vs. “forcing” of empirical data? A crucial problem of “grounded theory” reconsidered. Historical Social Research, Supplement.

[CR25] Kerner JF, Hall KL (2009). Research dissemination and diffusion translation within science and society. Research on Social Work Practice.

[CR26] Knight AT, Cowling RM, Rouget M, Balmford A, Lombard AT, Campbell BM (2008). Knowing but not doing: Selecting priority conservation areas and the research-implementation gap. Conservation Biology.

[CR28] Logan M (2010). Biostatistical design and analysis using R.

[CR29] Lomas J (1993). Diffusion, dissemination and implementation: Who should do what?. Annals New York Academy of Science.

[CR30] Matzek V, Covino J, Funk JL, Saunders M (2013). Closing the knowing-doing gap in invasive plant management: Accessibility and interdisciplinarity of scientific research. Conservation Letters.

[CR31] Meijaard E, Sheil D (2007). Is wildlife research useful for wildlife conservation in the tropics? A review for Borneo with global implications. Biodiversity and Conservation.

[CR32] Milner-Gulland EJ, Barlow J, Cadotte MW, Hulme PE, Kerby G, Whittingham MJ (2012). Ensuring applied ecology has impact. Journal of Applied Ecology.

[CR600] Moon K, Blackman D (2014). A guide to understanding social science research for natural scientists. Conservation Biology.

[CR33] Nadkarni NM (2004). Not preaching to the choir: Communicating the importance of forest conservation to nontraditional audiences. Conservation Biology.

[CR34] Ostrom E (2010). Polycentric systems for coping with collective action and global environmental change. Global Environmental Change.

[CR35] Painter RLE, Duran A, Miro E (2011). Indigenous alliances for conservation in Bolivia. Conservation Biology.

[CR36] Pietri DM, Gurney GG, Benitez-Vina N, Kuklok A, Maxwell SM, Whiting L, Vina MA, Jenkins LD (2013). Practical recommendations to help students bridge the research–implementation gap and promote conservation. Conservation Biology.

[CR37] Pullin AS, Knight TM (2009). Doing more good than harm—Building an evidence-base for conservation and environmental management. Biological Conservation.

[CR38] Quinn G, Keough M (2002). Experimental design and data analysis for biologists.

[CR39] R Core Development Team. 2013. R: a language and environment for statistical computing. Vienna, Austria: R Foundation for Statistical Computing. https://www.R-project.org/.

[CR40] Saldaña J (2015). The coding manual for qualitative researchers.

[CR41] SERNAP. 2012. *Conocimientos científicos y prioridades de investigación en el Parque Nacional y Área Natural de Manejo Integrado Madidi*. Eds. Salinas, E. and R. B. Wallace. La Paz, Bolivia: SERNAP.

[CR42] Shackleton CM, Cundill G, Knight AT (2009). Beyond just research: Experiences from southern Africa in developing social learning partnerships for resource conservation initiatives. Biotropica.

[CR43] Shanley P, Laird S, Laird SA (2002). ‘Giving back’: Making research results relevant to local groups and conservation. Biodiversity and traditional knowledge: Equitable partnerships in practice.

[CR44] Smith LT (1999). Decolonizing methodologies: Research and indigenous peoples.

[CR45] Sunderland T, Sunderland-Groves J, Shanley P, Campbell B (2009). Bridging the gap: How can information access and exchange between conservation biologists and field practitioners be improved for better conservation outcomes?. Biotropica.

[CR46] Sutherland WJ, Pullin AS, Dolman PM, Knight TM (2004). The need for evidence-based conservation. Trends in Ecology & Evolution.

[CR47] Toomey AH (2016). What happens at the gap between knowledge and practice? Spaces of encounter and misencounter between environmental scientists and local people. Ecology and Society.

[CR48] Toomey AH, Knight AT, Barlow J (2017). Navigating the space between research and implementation in conservation. Conservation Letters.

[CR49] Van Kerkhoff LE, Lebel L (2015). Coproductive capacities: Rethinking science-governance relations in a diverse world. Ecology and Society.

[CR50] Walsh JC, Dicks LV, Sutherland WJ (2014). The effect of scientific evidence on conservation practitioners’ management decisions. Conservation Biology.

[CR51] Zimmerer KS (2006). Cultural ecology: At the interface with political ecology—The new geographies of environmental conservation and globalization. Progress in Human Geography.

