# Supplementary material for: A question of dissemination: Assessing the practices and implications of research in tropical landscapes
Source: Ambio. 2018 Apr 24;48(1):35–47. doi: 10.1007/s13280-018-1056-5 (PMC6297105; doi:10.1007/s13280-018-1056-5)
Supplement: Supplementary file 1 — Supplementary material 1 (PDF 358 kb) [file 13280_2018_1056_MOESM1_ESM.pdf]

Ambio

Electronic Supplementary Material
This supplementary material has not been peer reviewed.

Title: A question of dissemination: Assessing the practices and implications of research in tropical landscapes

Authors: Anne H. Toomey, María Eugenia Copa Alvaro, Matthew Aiello-Lammens, Oscar Loayza, Jos Barlow

Appendix S1: Sample interview questions for semi-structured interviews with park guards

1. Do many researchers come to the park? What do they do? Do they always come by the central office? Do they ask the park guards for help (if so what kind)?
2. Have you had any experience working with or helping scientists or researchers in the park? What were they doing? What was your role (guide, informant, etc.?)
3. How was the experience for you? What did you learn?
4. Do the researchers that come involve the local communities in any way? How? (as guides, interpreters, etc.)?
5. How do you think local people perceive researchers?
6. How do researchers treat local people?
7. Do you think research is important? Why? What does it provide?
8. Do researchers disseminate locally the results of their work?
9. Do research results help to inform or influence the management of the protected area? If not, why do you think that might be? If yes, how? (Ask for examples)
10. How is information communicated between the park staff and the local population?
11. If there are results from a research project that would be important for local communities to know about (for example, the environmental impacts of constructing a road through the park), how would that information be communicated? Is there a process in place, or would one need to be created?
12. Do you believe it's important to disseminate technical information to local communities? Why or why not?
13. How do you think local people could be more involved in scientific research?
14. What types of research studies are lacking in the region?

15. What information does the park need to improve management and support conservation?

16. How do you think research could further support conservation in the region?

17. What would have to be improved or change in order to achieve that?

Appendix S2: Additional details on methodology

The research described in this paper is based on an applied methodology with an action research component, which was undertaken as part of a PhD based at the Lancaster Environmental Centre at Lancaster University. The main objective of the PhD research was to study the gap between conservation research and practice in the Madidi NP/NAIM and surrounding region, which was done by taking a closer look on which what information natural resources management decisions were based, and specifically the role of scientific research and researchers in these processes. Key to this understanding was who makes decisions and the values and worldviews on which these decisions are based.

This research was carried out during four key periods in Bolivia, including a reconnaissance visit January-February of 2012 (one month), a preliminary fieldwork period June-December of 2012 (six months), a main fieldwork stage May-December 2013 (6.5 months), and a period of validation of data/preliminary dissemination stage June-August in 2014 (2 months), which included some documentary filming of interviews previously conducted. A final dissemination stage was carried out between September-November in 2015 (2.5 months) with all of the involved participants/actors mentioned in the research.

Workshops

Workshops were carried out with three stakeholder groups – park guards, indigenous communities, and scientists. In the case of the park guards, the workshops were structured in part around the systematic analysis of past research conducted in the protected area. The main aim was to discuss what had been done previously and to what extent those studies had been disseminated/ implemented for management, and in the

case of Madidi, to develop a specific regulation for research in the protected area. These questions led to wider discussions about personal experiences with research and about the relevance of research in general. Workshops were also held in two ‘over-researched’ indigenous communities, and were organized around a proposal to create a community norm to negotiate their relations with researchers in the future (i.e. ethical protocols). Between 2014-2015 these norms were revisited with local leaders, and although they were said to provide a ‘reference’ for the communities with regards to research, their usefulness was very limited. A more effective approach would be longer-term and community-led, but this was not possible due to the time and budgetary constraints of the PhD, in addition to the fact that the communities had much more pressing issues to deal with at the time. Two ‘communication and dissemination training’ workshops were also held with students and staff of the National Herbarium (a botanical institute) in La Paz. These were developed and carried out by request of the Herbarium’s director, and attended by 40+ participants. Activities included individual and collective reflection, role play and group discussion. Between December of 2012 and August of 2014 feedback sessions were held with: Madidi NP/NAIM, a group of Bolivian researchers from various institutions, and the Takana, San Jóse de Uchupiamonas, and Tsimane’-Mosetén indigenous councils. Additional meetings to present findings and materials were held with the Wildlife Conservation Society, Pilón Lajas BR/IT, SERNAP, and the Vice-Ministry of the Environment’s Department of Biodiversity.

Interviews

Interviews were focused on understanding the main issues involved with scientific research and the management of natural resources in the Madidi region, specifically

regarding experiences with park management, main concerns of community regarding conservation activities in the region, and awareness of existing scientific research and monitoring. Most interviews lasted between 20-40 minutes, but some discussions with key individuals continued over many hours or even days due to close proximity. Other interviews were conducted very briefly (10-15 minutes) to ask about a person's specific perception of a given situation or project. Questions focused on how decisions about natural resources use are made at local, regional and national levels, the role of scientific research in those decisions, micro and macro-level politics and encounters around the production and dissemination of scientific research. Some of these interviews were also repeated and filmed in 2014, and two short documentaries were created as a result, Bolivian Perspectives on Research, and the Park Guards of Madidi, both of which are publically available on YouTube (https://youtu.be/uQd95Nq05Rk and https://youtu.be/H-ZuJccV4as). Questions and the discussions (and duration) varied according to the situation and interviewee, but a sample interview schedule is provided in Appendix S1.

Appendix S3: Code List with frequencies (*n* of quotations associated with this code). Codes that were deemed highly relevant are highlighted in grey, and the quotations for these codes were compiled and re-analysed.

**Specific codes**	**Code frequency**
Changing ideas/ worldviews/ knowledge	5
Diffusion and Dissemination	56
Community decision-making	17
Different values/ worldviews	18
Ethical research issues	13
Legality / regulations	5
Local participation in science	23
Local perceptions of research	64
Management / Conservation	23
Monitoring	15
Obtaining consent	23
Research-implementation gap	16
Research difficulties	8
Research power issues	25
Research priorities in Madidi	20
Results / *recuerdos* / Benefits	34
Researcher self-reflexivity	4
Spaces of encounter	23
Technical or scientific information	11
Knowledge integration debates	19
What is research?	11
